# Species conservation profile and updated assessment of the genus *Nototriche* Turcz. (Malvaceae, Malvoideae) in the páramos of Ecuador

**DOI:** 10.3897/BDJ.14.e203556

**Published:** 2026-10-23

**Authors:** Emilio J. Trujillo, Priscilla Muriel

**Affiliations:** 1 Herbario QCA, Centro de Investigaciones de la Biodiversidad CIBIO, Facultad de Ciencias Exactas, Naturales y Ambientales, Pontificia Universidad Católica del Ecuador, Quito, Ecuador Herbario QCA, Centro de Investigaciones de la Biodiversidad CIBIO, Facultad de Ciencias Exactas, Naturales y Ambientales, Pontificia Universidad Católica del Ecuador Quito Ecuador

**Keywords:** Andes, conservation, endangered, IUCN, *
Nototriche
*, páramo

## Abstract

**Background:**

The high-Andean Malvaceae of Ecuador is represented by a single genus, *Nototriche* Turcz. Currently, the genus comprises five species distributed throughout the country, across the Andes mountain range, in ecosystems such as páramo and superpáramo. These high-Andean ecosystems have been the focus of conservation efforts because of their ecological fragility, high diversity and the large number of specialised plants adapted to them. Although two conservation assessments have been completed for Ecuadorian *Nototriche*, the most recent ones 21 and 14 years ago, these have not been processed through the official IUCN Red List. Consequently and considering the recent description of a new taxon for the genus, a full reassessment for the group in the country is therefore needed.

**New information:**

We present a re-evaluation for two species and new conservation assessments for three species of the five accepted *Nototriche* representatives in Ecuador. *Nototriche antisanensis* E.J. Trujillo, Muriel, Espinel-Ortiz & Romol., *Nototriche hartwegii* A.W.Hill and *Nototriche phyllanthos* (Cav.) A.W.Hill are completely assessed officially for the first time and we also re-evaluate *Nototriche ecuadoriensis* Fryxell and *Nototriche jamesonii* A.W.Hill after 22 years due to changes in the real distribution of the species and populations fluctuations. This work illustrates the need for new research on páramo vegetation in the Ecuadorian Andes.

## Introduction

Amongst all alpine ecosystems, the páramos are the most biodiverse areas regarding vascular plants, having a high rate of speciation and endemism (Pérez-Escobar et al. 2022). This ecosystem is characterised by extreme climatic conditions, having high temperatures throughout day and reaching below zero temperatures at night, leading to the biota that lives there to generate several mechanisms of adaptation to establish, survive and reproduce in such variable conditions (Sarmiento 1986, Sklenář et al. 2015). Despite their remarkable biodiversity, the páramos remain inadequately sampled, with substantial gaps in specimen curation, species discovery and distributional knowledge, limiting efforts to overcome the Linnean and Wallacean shortfalls (Lomolino et al. 2005). In Ecuador, a study published by Romoleroux et al. (2023) documented 1735 páramo plant species throughout the country, of which 690 are endemic to this ecosystem. Amongst the endemic species, approximately, 75% were categorised as threatened in 2011 national Red List assessment (León-Yánez et al. 2011), highlighting the urgent need for updated conservation assessments.

*Nototriche* species are herbs, prostrate shrubs or cushions that form lax or dense rosettes of basal leaves, which can be shortly caulescent. The stems are woody, sometimes with several branches and can grow in soil, wind-blown dust or sand. The leaves vary in shape, but in Ecuador, they share a pinnati- or palmati-lobed form with five or more lobes. The stipules are adnate to the petiole, forming a protective sheath. Flowers are solitary and sometimes the pedicel arises at the centre of the petiole. Fruits are schizocarps densely covered by various types of indumentum (Hill 1909, Krapovickas 1951, Fryxell 1992, Mazzei 2025).

In South America, *Nototriche* species are distributed from Ecuador at the north, to Argentina in the south (Mazzei 2025), but the genus might reach Colombian páramos. In Ecuador, the genus grows almost throughout all the páramos of the country, having records in the southern páramos of the Azuay Province and also at the northern páramos of Imbabura volcano, but the area of presence differs amongst species. Additionally, the Andean Cordillera in Ecuador is divided into two well-separated chains, the Eastern and Western mountain ranges and *Nototriche* is present in both. The species assessed in this study are endemic to Ecuador and grow only in high altitudes, between 4100 and 5000 m (Sklenář and Ramsay 2001). This elevational range corresponds to grass páramo and the superpáramo, the uppermost páramo belt situated just below the permanent snowline. Unlike the grass páramo proper, dominated by continuous tussock grasslands, the superpáramo is characterised by open, discontinuous vegetation over largely bare substrate (rock, sand and cryoturbated soils), with prostrate herbs, cushion plants and lichens (Sklenář and Ramsay 2001).

The conservation status of *Nototriche* in Ecuador has been evaluated at the national level on two previous occasions, although neither constitutes an officially registered assessment on the IUCN Red List. The first was made by Chanco and Ulloa-Ulloa (2004), who recognised four species for the country: *Nototriche ecuadoriensis* Fryxell, *N. hartwegii* A.W.Hill, *N. jamesonii* A.W.Hill and *N. phyllanthos* (Cav.) A.W.Hill; categorising *N. ecuadoriensis* and *N. hartwegii* as Endangered (EN) and *N. jamesonii* and *N. phyllanthos* as Least Concern (LC). Subsequently, León-Yánez et al. (2011), in the *Libro rojo de las plantas endémicas del Ecuador*, retained the categories established in 2004. Both evaluations, however, relied solely on herbarium specimens and previously published collection data, without updated field sampling or spatial analyses. To date, the only officially registered conservation assessments for the Ecuadorian species of *Nototriche* are those published on the IUCN Red List website by Montúfar and Pitman (2004a), Montúfar and Pitman (2004b).

In 2023, we started a project addressing the systematics of the genus *Nototriche* in Ecuador. This involved several field trips throughout the country's páramos, during which we sampled all the species, made new collections and updated the genus' distribution in Ecuador. The present study aims to provide an updated conservation work focusing on these species, given the lack of official assessments and the new valuable taxonomic and distributional data obtained during these last decades.

## Materials and Methods

We gathered distribution data from three Ecuadorian herbaria (QCA, QCNE and HA) and also extracted occurrence records from the Global Biodiversity Information Facility (https://www.gbif.org/es/) for all *Nototriche* species. Additionally and part of a systematic study of the genus, we made several field trips throughout the páramos of Ecuador to make new collections and obtain more geographical and ecological data for the genus to complete a comprehensive sampling of the taxon.

To determine the geographic distribution of the species, we reviewed the collected data, corrected wrong distribution points and imported the information into QGIS 3.36.2 (QGIS Development Team 2026) to generate the multiple distribution maps enclosing Ecuador. For geospatial analysis and the estimation of AOO and EOO, we used the software GeoCAT (Bachman et al. 2011) following the reccomendations of the IUCN. Finally, for the conservation assessments and re-evaluations, we followed the UICN Red List Categories and Criteria to assign a category (IUCN 2012).

## Results

We present an exhaustive updated conservation assessment for the genus in the country, re-evaluating two species and assessing officially three for the first time. Some localities mentioned in literature as recording collections of several species, records that had influenced the previous evaluations, were disregarded, based on our fieldwork and the herbarium work that, with the new identification, changed fundamental metrics in the conservation analyses. Following the re-evaluation of the two species, we propose that *N. ecuadoriensis* and *N. jamesonii* be reclassified as Critically Endangered and Endangered, respectively. In addition, we suggest that *N. antisanensis* be treated as Critically Endangered, *N. hartwegii* as Endangered and *N. phyllanthos* as Vulnerable.

All these results are summarised in Table 1.

## Species Conservation Profiles

### Nototriche antisanensis

#### Species information

Scientific name: Nototriche antisanensis

Species authority: E.J. Trujillo, Muriel, Espinel-Ortiz & Romol.

Synonyms: No synonyms have been registered for this species.

Common names: No common names have been registered for this species.

Kingdom: Plantae

Phylum: Tracheophyta

Class: Magnoliopsida

Order: Malvales

Family: Malvaceae

Taxonomic notes: *Nototriche antisanensis* is the most recently described species of the genus for Ecuador. It can be recognised by the combination of the following traits: palmati-lobed leaves with short triangular stipules and presence of stellate trichomes in both faces of the leaf blade, connate petals forming a short corolla tube and a cushion habit.

Figure(s) or Photo(s): Fig. 1

Region for assessment: Global

#### Editor & Reviewers

##### Reviewers

Reviewers: N/A

##### Editor

Editor: Trujillo EJ, Muriel P

#### Geographic range

Biogeographic realm: Neotropical

Countries: Ecuador

Map of records (image): Fig. 2

Map of records (Google Earth): None

Basis of EOO and AOO: Observed

Basis (narrative): The extent of occurrence (EOO) is 0.03 km and the maximum actual area of occupancy (AOO) is 8.00 km.

Min Elevation/Depth (m): 4325

Max Elevation/Depth (m): 4325

Range description: Endemic species to Ecuador. It has been only recorded at its type locality in the Antisana Ecological Reserve, at the north-eastern part of the mountain range.

#### Extent of occurrence

EOO (km2): 0.03

Trend: Unknown

Justification for trend: Although it is a small population, the area where it occurs on Antisanilla Peak is not heavily visited by humans, nor is it directly affected by land transformation. However, given its small size, the population is extremely fragile and the number of individuals could decline rapidly as a result of land-cover changes driven by land-use and climate threats.

Causes ceased?: Unknown

Causes understood?: Unknown

Causes reversible?: Unknown

Extreme fluctuations?: Unknown

#### Area of occupancy

Trend: Unknown

Justification for trend: Similar to the EOO, there is not enough evidence to establish a concrete trend. The populations need monitoring throughout the coming years to identify a trend.

Causes ceased?: Unknown

Causes understood?: Unknown

Causes reversible?: Unknown

Extreme fluctuations?: Unknown

AOO (km2): 8.00

#### Locations

Number of locations: 1

Justification for number of locations: It is only known from its type locality at Antisanilla Peak around Antisana volcano.

Trend: Unknown

Justification for trend: At present, the locality experiences little anthropogenic intervention and lies within a government protected reserve. However, the ecosystem to which it belongs is extremely fragile in the face of human activity: throughout its history, the Antisana volcano has been affected by the expansion of the agricultural frontier and by cattle ranching.

Extreme fluctuations?: No

#### Population

Number of individuals: Estimated less than 50 mature individuals.

Trend: Unknown

Justification for trend: The number of individuals is critically low and we do not have any evidence to establish a consistent trend. However, this could change in the following years due to climatic threats or anthropogenic activities.

Causes ceased?: Unknown

Causes understood?: Unknown

Causes reversible?: Unknown

Extreme fluctuations?: Unknown

#### Subpopulations

Trend: Unknown

Extreme fluctuations?: Unknown

Severe fragmentation?: Unknown

#### Habitat

System: Terrestrial

Habitat specialist: Yes

Habitat (narrative): This species occurs in superpáramos (above the páramo line, this case at 4,325 m), which are characterised by strong adiabatic winds, sandy soils, high exposure to abiotic conditions like sun and rain and have scattered vegetation.

Trend in extent, area or quality?: Decline (observed)

Justification for trend: A slow, but continous decline of this habitat has been recorded through time, being affected by the expansion of the agricultural frontier and, moreover, vulnerable to Climate Change (CC) (Hofstede et al. 2023a). A small increase of temperature in these habitats will drastically affect the ecosystemic composition, causing a shifting in vegetation coverture (Hofstede et al. 2023b) and the reduction of the habitat were N. antisanensis grows.

##### Habitat

Habitat importance: Major Importance

Habitats: 6. Rocky areas (e.g. inland cliffs, mountain peaks)

#### Ecology

Size: 30 cm long, 30 cm wide, height of 40 cm.

Generation length (yr): 0

Dependency of single sp?: No

Ecology and traits (narrative): As noted above, *Nototriche antisanensis* predominantly occurs on sandy substrates with sparse vegetation dominated by members of Poaceae and Fabaceae. Due to its cushion growth form, several small vascular species are commonly found growing amongst the compact rosettes of *N. antisanensis*. The roots are long and firmly anchored in the substrate, an apparent adaptation to access subsurface moisture in these well-drained soils.

#### Threats

Justification for threats: Although *Nototriche antisanensis* occurs inside the Antisana National Park and thus benefits from legal protection, its habitat having long experienced human pressure, agricultural expansion, livestock grazing, government water infrastructure projects and increasing recreational use tied to tourism. In addition, regional climate change has produced measurable shifts in temperature: the páramo surrounding Antisana is undergoing anomalous warming (Moret et al. 2019) and the vegetation there is already responding to these changes. Together, land-use pressures and climatic shifts represent persistent threats to the long-term survival of *N. antisanensis*.

##### Threats

Threat type: Ongoing

Threats: 2.1.2. Agriculture & aquaculture - Annual & perennial non-timber crops - Small-holder farming2.1.4. Agriculture & aquaculture - Annual & perennial non-timber crops - Scale Unknown/Unrecorded2.3.2. Agriculture & aquaculture - Livestock farming & ranching - Small-holder grazing, ranching or farming3.3. Energy production & mining - Renewable energy6.1. Human intrusions & disturbance - Recreational activities10.1. Geological events - Volcanoes11.1. Climate change & severe weather - Habitat shifting & alteration11.2. Climate change & severe weather - Droughts11.3. Climate change & severe weather - Temperature extremes11.5. Climate change & severe weather - Other impacts

##### Threats

Threat type: Future

Threats: 11.1. Climate change & severe weather - Habitat shifting & alteration11.2. Climate change & severe weather - Droughts11.3. Climate change & severe weather - Temperature extremes11.5. Climate change & severe weather - Other impacts

#### Conservation

Justification for conservation actions: Although the Antisana National Park confers formal protection to the localities where *Nototriche antisanensis* occurs, no species-specific conservation measures have been implemented yet; this study represents the first complete conservation assessment of *N. antisanensis* since its description in 2025 and thus fills a critical knowledge gap. Immediate complementary actions are required to secure the species long-term persistence: raising local awareness through targeted outreach and environmental education, explicitly incorporating *N. antisanensis* into the park management plan and into any water-infrastructure planning, implementing habitat-level protection plans and active restoration of degraded páramo patches, establishing long-term population and climate-impact monitoring and considering ex-situ methodologies (e.g. seed banking or living collections) alongside research on population dynamics and reproductive biology.

##### Conservation actions

Conservation action type: In Place

Conservation actions: 1.1. Land/water protection - Site/area protection

##### Conservation actions

Conservation action type: Needed

Conservation actions: 2.3. Land/water management - Habitat & natural process restoration4.3. Education & awareness - Awareness & communications

#### Other

##### Use and trade

Use type: International

### Nototriche ecuadoriensis

#### Species information

Scientific name: Nototriche ecuadoriensis

Species authority: Fryxell

Synonyms: No synonyms have been registered for this species.

Common names: No common names have been registered for this species.

Kingdom: Plantae

Phylum: Tracheophyta

Class: Magnoliopsida

Order: Malvales

Family: Malvaceae

Taxonomic notes: *Nototriche ecuadoriensis* can be easily recognised by the following combination of traits: pinnately lobed leaves with long triangular stipules ending with an obtuse apex, a glabrous leaf blade, except for a few ciliate trichomes at the lobe apices, connate petals forming a long corolla tube and an acaulescent herbaceous habit.

Figure(s) or Photo(s): Fig. 3

Region for assessment: Global

#### Editor & Reviewers

##### Reviewers

Reviewers: N/A

##### Editor

Editor: Trujillo EJ, Muriel P

#### Geographic range

Biogeographic realm: Neotropical

Countries: Ecuador

Map of records (image): Fig. 4

Map of records (Google Earth): None

Basis of EOO and AOO: Observed

Basis (narrative): The extent of occurrence (EOO) is 243.50 km and the maximum actual area of occupancy (AOO) is 16.00 km .

Min Elevation/Depth (m): 4050

Max Elevation/Depth (m): 4567

Range description: Endemic to Ecuador, this species has only been recorded in two localities: on the western slopes of Antisana volcano and within the Cayambe-Coca National Park, both in the north-eastern Andes of the country.

#### Extent of occurrence

EOO (km2): 243.50

Trend: Unknown

Justification for trend: There is not enough evidence to determine a trend in the extent of occurrence. The areas where this species grows are threatened and may undergo reduction, but such changes are difficult to detect due to the area's small size.

Causes ceased?: Unknown

Causes understood?: Unknown

Causes reversible?: Unknown

Extreme fluctuations?: Unknown

#### Area of occupancy

Trend: Unknown

Justification for trend: Based in our observations, the area of occupancy of *N. ecuadoriensis* does not present reduction. However, the evidence is not enough to determinate that the trend is "stable" and, as mentioned earlier, the populations present several threats.

Causes ceased?: Unknown

Causes understood?: Unknown

Causes reversible?: Unknown

Extreme fluctuations?: Unknown

AOO (km2): 16.00

#### Locations

Number of locations: 2

Justification for number of locations: This species is found in two separated localities: around Cayambe-Coca National Park and around Antisana volcano. Both locations are separated form one another by approximately 20 km.

Trend: Unknown

Justification for trend: Both locations where *N. ecuadoriensis* grows are inside protected reserves. However, there is not enough evidence to establish a concrete trend due to the active threats the area is experimenting, in spite of its protection status.

Extreme fluctuations?: Unknown

#### Population

Number of individuals: Predicted less than 50 mature individuals.

Trend: Decline (observed)

Justification for trend: Although we do not know the actual or projected number of individuals in the entire population, in one locality (the type locality), the population of *N. ecuadoriensis* has declined, as no individuals were found at the coordinates where it was originally described.

Basis for decline: (a) direct observation

Causes ceased?: Unknown

Causes understood?: Unknown

Causes reversible?: Unknown

Extreme fluctuations?: Unknown

#### Subpopulations

Number of subpopulations: 3

Trend: Decline (observed)

Justification for trend: The subpopulations found at the Cayambe Coca National Park have declined and the individuals have being replaced by other herbaceous plants. Moreover, this species has not been found at the type locality in recent years, showing clear evidence of a reduction in the number of individuals. The subpopulation at the western flank of Antisana volcano is stable, probably because it is located in an area with difficult accessibility.

Extreme fluctuations?: Unknown

Severe fragmentation?: Yes

#### Habitat

System: Terrestrial

Habitat specialist: Yes

Habitat (narrative): *N. ecuadoriensis* grows in páramos with abundant herbaceous vegetation and scattered shrubby plants. They are characterised by high humidity in air and soil, strong winds and low temperatures throughout the day and at night.

Trend in extent, area or quality?: Decline (observed)

Justification for trend: The humid páramos where this species is recorded have experienced a decline in area and have become degraded due to anthropogenic activities like road building (one of the principal connections to the Amazon Region) and tourism (hot springs, fishing, trekking etc.).

##### Habitat

Habitat importance: Major Importance

Habitats: 6. Rocky areas (e.g. inland cliffs, mountain peaks)

#### Ecology

Size: 10 cm long, 7 cm wide, 5 cm height and 6 cm of underground length (all aproximate values).

Generation length (yr): 0

Dependency of single sp?: No

Ecology and traits (narrative): *Nototriche ecuadoriensis* prefers to grow in humid conditions, in association with other plant taxa like *Azorella*, *Diplostephium* or *Valeriana*. It is normally found between other plants probably to protect reproductive structures such as flowers, buttons and fruits.

#### Threats

Justification for threats:

Although both areas where this species grows are under protected reserve schemes, we identified several threats that could affect its populations, reduce the number of individuals and alter or affect its growing area. The construction of roads connecting the Amazon Region with the Andean Region represents a threat to the species; these roads are also followed by oil pipelines and tourism activities associated with the hot springs in the area are present along them as well. Anthropogenic activities, like agriculture and cattle raising, are inevitable. Additionally, pollution and natural system modifications exist, affecting areas nearby.

Finally, both localities are situated within the range of Antisana's most dangerous volcanic activities (pyroclastic flow, lahars, ashes and lava flow, see Hall et al. (2012)) and are exposed to climate driven threats, like temperature rise associated with global warming, all which are expected to reduce the extent of characteristic páramo vegetation.

##### Threats

Threat type: Ongoing

Threats: 1.1. Residential & commercial development - Housing & urban areas1.3. Residential & commercial development - Tourism & recreation areas2.1.2. Agriculture & aquaculture - Annual & perennial non-timber crops - Small-holder farming3.3. Energy production & mining - Renewable energy4.1. Transportation & service corridors - Roads & railroads6.1. Human intrusions & disturbance - Recreational activities7.2. Natural system modifications - Dams & water management/use7.2.1. Natural system modifications - Dams & water management/use - Abstraction of surface water (domestic use)7.2.2. Natural system modifications - Dams & water management/use - Abstraction of surface water (commercial use)7.2.3. Natural system modifications - Dams & water management/use - Abstraction of surface water (agricultural use)7.2.4. Natural system modifications - Dams & water management/use - Abstraction of surface water (unknown use)9.1.1. Pollution - Domestic & urban waste water - Sewage9.1.2. Pollution - Domestic & urban waste water - Run-off9.4. Pollution - Garbage & solid waste10.1. Geological events - Volcanoes11.1. Climate change & severe weather - Habitat shifting & alteration11.3. Climate change & severe weather - Temperature extremes11.5. Climate change & severe weather - Other impacts

##### Threats

Threat type: Future

Threats: 11.1. Climate change & severe weather - Habitat shifting & alteration11.2. Climate change & severe weather - Droughts11.3. Climate change & severe weather - Temperature extremes11.5. Climate change & severe weather - Other impacts

#### Conservation

Justification for conservation actions: There is not any specific action to conserve *N. ecuadoriensis*; however, it grows inside a protected reserve that can slow down the processes of land-change and ecosystem loss.

##### Conservation actions

Conservation action type: In Place

Conservation actions: 1.1. Land/water protection - Site/area protection

##### Conservation actions

Conservation action type: Needed

Conservation actions: 2.3. Land/water management - Habitat & natural process restoration4.3. Education & awareness - Awareness & communications

#### Other

##### Use and trade

Use type: International

##### Ecosystem services

Ecosystem service type: Very important

### Nototriche hartwegii

#### Species information

Scientific name: Nototriche hartwegii

Species authority: A.W. Hill

Synonyms: No synonyms have been registered for this species.

Common names: No common names have been registered for this species.

Kingdom: Plantae

Phylum: Tracheophyta

Class: Magnoliopsida

Order: Malvales

Family: Malvaceae

Taxonomic notes: This species can be recognised by the combination of the following traits: the large palmati-lobed leaves, long lanceolate stipules, a high density of long stellate trichomes in both faces of the leaf blade, free petals and a prostrate shrub or cushion habit, depending on locality and abiotic conditions.

Figure(s) or Photo(s): Fig. 5

Region for assessment: Global

#### Editor & Reviewers

##### Reviewers

Reviewers: N/A

##### Editor

Editor: Trujillo EJ, Muriel P

#### Geographic range

Biogeographic realm: Neotropical

Countries: Ecuador

Map of records (image): Fig. 6

Map of records (Google Earth): None

Basis of EOO and AOO: Observed

Basis (narrative): The extent of occurrence (EOO) is 10492.00 km and the maximum actual area of occupancy (AOO) is 28.00 km.

Min Elevation/Depth (m): 4310

Max Elevation/Depth (m): 4545

Range description: Species endemic to Ecuador. It is the most widely distributed species of the genus in the country, ocurring in fragmented locations from the southern to the northern border.

#### Extent of occurrence

EOO (km2): 10492.00

Trend: Unknown

Justification for trend: There is not enough evidence to establish a trend in the EOO for *N. hartwegii*. The localities where this species grows are very different from each other.

Causes ceased?: Unknown

Causes understood?: Unknown

Causes reversible?: Unknown

Extreme fluctuations?: Unknown

#### Area of occupancy

Trend: Unknown

Justification for trend: There is no evidence to establish a trend regarding the area of occupancy, due to the fragmentation and distance between the different locations and their unknown number of individuals, in addition to the lack of monitoring efforts.

Causes ceased?: Unknown

Causes understood?: Unknown

Causes reversible?: Unknown

Extreme fluctuations?: Unknown

AOO (km2): 28.00

#### Locations

Number of locations: 5

Justification for number of locations: There are five known localities for this species: Cajas National Park in the south, Páramos of Ozogoche and Quispicacha in the central Andes and Imbabura volcano in northern Ecuador.

Trend: Unknown

Justification for trend: The trend for the number of locations cannot be generalised due to the large differences between them. The southern and central locations are protected under reserves; therefore, the number of locations could be maintained in the future. However, the northern locality is not under any protection scheme, which makes it difficult to assess if the number of locations would increase or diminish in the zone.

Extreme fluctuations?: Unknown

#### Population

Number of individuals: Unknown, but we estimated less than 100 mature individuals.

Trend: Unknown

Justification for trend: There is not enough evidence to establish a trend. More monitoring is necessary.

Causes ceased?: Unknown

Causes understood?: Unknown

Causes reversible?: Unknown

Extreme fluctuations?: Unknown

#### Subpopulations

Number of subpopulations: 5

Trend: Unknown

Justification for trend: As all the subpopulations are hetergenous between them, there is no rigorous monitoring process that gives us enough evidence to establish a trend. However, due to changes in land-cover and ecosystem conversion, we can infer that the subpopulations are severely fragmented and with large distance from one another, approximately, 93.03 to 165.19 km, making gene flow technically impossible.

Extreme fluctuations?: Unknown

Severe fragmentation?: Yes

#### Habitat

System: Terrestrial

Habitat specialist: Yes

Habitat (narrative): *N. hartwegii* grows in páramos dominated by different characteristics depending on their location. In southern Ecuador, the species grows in sandy soils at mountain slopes with scattered vegetation, protected from abiotic conditions like strong winds present on the opposite side of the Andean chain. Around the central Andes, it grows near the surroundings of more humid places like wetlands. Finally, at the northern part of the country it also grows in mountain slopes, characterised by humid soils with dense vegetation and exposed to strong winds and fog.

Trend in extent, area or quality?: Decline (observed)

Justification for trend: Despite four of the five localities are inside a national park or protected area, we observed a decline in the quality and area of the habitat where *N. hartwegii* grows due to expansion of the agricultural frontier, farming and road building. Additionally, touristic activites like trekking or popular viewpoints are very near some populations (at Cajas National Park at South or Imbabura volcano at north).

##### Habitat

Habitat importance: Major Importance

Habitats: 6. Rocky areas (e.g. inland cliffs, mountain peaks)

#### Ecology

Size: 30 cm length, 40 cm width, height of 8 cm and underground length unknown due to its habit (all aproximate values).

Generation length (yr): 0

Dependency of single sp?: No

Ecology and traits (narrative): *Nototriche hartwegii* can grow under different conditions, being found in both dry and humid climates. In drier habitats, it typically forms compact leaf rosettes, whereas in more humid conditions, the rosettes are more loosely arranged. In some populations, the species also forms cushion-like growth structures that facilitate the establishment of other plant species within its canopy, a growth form similar to that observed in *N. antisanensis*.

#### Threats

Justification for threats: As other *Nototriche* species in Ecuador, *N. hartwegii* grows in páramos and superpáramos, which are extremely delicate ecosystems and that have suffered intense degradation for centuries, due to anthropogenic influence. Human activities that affect these ecosystems are: road building, agriculture, farming, establishment of energy projects, recreational activites and pollution. Additionally, global issues such as climate change affect in a stronger manner these fragile areas, due to the effect elevation has on precipitation and temperature changes (Sklenář et al. 2021).

##### Threats

Threat type: Ongoing

Threats: 1.1. Residential & commercial development - Housing & urban areas1.3. Residential & commercial development - Tourism & recreation areas2.1.2. Agriculture & aquaculture - Annual & perennial non-timber crops - Small-holder farming3.3. Energy production & mining - Renewable energy4.1. Transportation & service corridors - Roads & railroads6.1. Human intrusions & disturbance - Recreational activities7.3. Natural system modifications - Other ecosystem modifications9.1.3. Pollution - Domestic & urban waste water - Type Unknown/Unrecorded9.3.4. Pollution - Agricultural & forestry effluents - Type Unknown/Unrecorded9.4. Pollution - Garbage & solid waste11.3. Climate change & severe weather - Temperature extremes11.5. Climate change & severe weather - Other impacts

##### Threats

Threat type: Future

Threats: 11.1. Climate change & severe weather - Habitat shifting & alteration11.2. Climate change & severe weather - Droughts11.3. Climate change & severe weather - Temperature extremes11.5. Climate change & severe weather - Other impacts

#### Conservation

Justification for conservation actions: There is not any specific action to conserve *N. hartwegii*; however, most of the populations grow inside protected areas or reserves (Parque Nacional Cajas, Reserva de Producción Faunística Chimborazo, Parque Nacional Sangay) that can slow down the impact of threats on this species.

##### Conservation actions

Conservation action type: In Place

Conservation actions: 1.1. Land/water protection - Site/area protection

##### Conservation actions

Conservation action type: Needed

Conservation actions: 2.3. Land/water management - Habitat & natural process restoration4.3. Education & awareness - Awareness & communications

### Nototriche jamesonii

#### Species information

Scientific name: Nototriche jamesonii

Species authority: A.W. Hill

Synonyms: = *Nototriche chimborazoensis* Hochr.

Common names: No common names have been registered for this species.

Kingdom: Plantae

Phylum: Tracheophyta

Class: Magnoliopsida

Order: Malvales

Family: Malvaceae

Taxonomic notes: *Nototriche jamesonii* can be recognised by the combination of the following traits: palmati-lobed leaves with several lobe subdivisions (> 9), long triangular stipules, both faces of the leaf blade covered by small stellate trichomes, connate petals forming a corolla tube (longer than *N. antisanensis*, but shorter than *N. ecuadoriensis*) and a prostrate shrub habit.

Figure(s) or Photo(s): Fig. 7

Region for assessment: Global

#### Editor & Reviewers

##### Reviewers

Reviewers: N/A

##### Editor

Editor: Trujillo EJ, Muriel P

#### Geographic range

Biogeographic realm: Neotropical

Countries: Ecuador

Map of records (image): Fig. 8

Map of records (Google Earth): None

Basis of EOO and AOO: Observed

Basis (narrative): The extent of occurrence (EOO) is 712.15 km and the maximum actual area of occupancy (AOO) is 48.00 km^2^.

Min Elevation/Depth (m): 3800

Max Elevation/Depth (m): 4962

Range description: Species endemic to Ecuador, restricted to the Ecuadorian central Andes, having the highest concentration of individuals occurring on the western slopes Chimborazo volcano.

#### Extent of occurrence

EOO (km2): 712.15

Trend: Unknown

Justification for trend: There is not enough evidence to establish a trend in the extent of occurrence. More monitoring and biological sampling periodically is needed.

Causes ceased?: Unknown

Causes understood?: Unknown

Causes reversible?: Unknown

Extreme fluctuations?: Unknown

#### Area of occupancy

Trend: Unknown

Justification for trend: We need more evidence to establish a trend in the area of occupancy for *N. jamesonii*. This species is well distributed around Chimborazo volcano, with several individuals, but more monitoring and sampling throughout the next years are necessary.

Causes ceased?: Unknown

Causes understood?: Unknown

Causes reversible?: Unknown

Extreme fluctuations?: Unknown

AOO (km2): 48.00

#### Locations

Number of locations: 2

Justification for number of locations: There are only two confirmed locations where this species has been recorded. In previous taxonomic revisions (Fryxell 1992, Chanco and Ulloa-Ulloa 2004), the distribution of this species was also extended to the northern part of the country. However, a recent revision by the authors (Trujillo et al., in prep.) showed that several specimens housed at QCA and QCNE had been misidentified as *N. jamesonii*. After curating these collections, we now restrict the species to the central Andes around Chimborazo volcano, with an additional record from Carihuairazo volcano.

Trend: Unknown

Justification for trend: Despite the curation of herbaria data and direct observation throughout fieldwork, to determine a trend in the number of locations, we need more sampling in other páramos like Carihuairazo volcano, Ilinizas volcanoes or Corazón volcano.

Extreme fluctuations?: Unknown

#### Population

Number of individuals: Unknown, but we estimated less than 2500 mature individuals.

Trend: Unknown

Justification for trend: *Nototriche jamesonii* is relatively abundant around Chimborazo volcano, with several individuals growing in sandy soils. However, the real number of individuals is unknown and, from our field observations, we estimate less than 2500 individuals grow in the volcano. More evidence is needed to establish a trend in the population.

Causes ceased?: Unknown

Causes understood?: Unknown

Causes reversible?: Unknown

Extreme fluctuations?: Unknown

#### Subpopulations

Trend: Unknown

Justification for trend: Just one subpopulation, the one around Chimborazo páramos, is well known. The other subpopulation, in Quispicacha, is known through several herbarium collections and one-day observations. A trend cannot be established yet; more evidence, monitoring and sampling are needed.

Extreme fluctuations?: Unknown

Severe fragmentation?: Yes

#### Habitat

System: Terrestrial

Habitat specialist: Yes

Habitat (narrative): *Nototriche jamesonii* only grows on dry sandy soils with scattered vegetation, characterstic of the superpáramo ecosystem. This species has a prostrate shrub habit, with several ramifications of the branches underground and highly compacted leaves. This harsh type of páramo (superpáramo) is similar to the Puna found in Perú, being a dry landscape with strong winds, poor precipitation and dominated by highly adapted plant species.

Trend in extent, area or quality?: Decline (observed)

Justification for trend: As many other páramos in Ecuador, the habitat where *N. jamesonii* grows is affected by anthropogenic activites, specially by tourism. Chimborazo, the tallest of all Ecuadorian volcanoes, is one of the most famous peaks throughout the country. Therefore, it serves as a heavily visited touristic attraction. This has an important impact on the flora, due to construction of roads, pollution and trampling of the vegetation related to trekking activites.

##### Habitat

Habitat importance: Major Importance

Habitats: 6. Rocky areas (e.g. inland cliffs, mountain peaks)

#### Ecology

Size: 20 cm length, 15 cm width, height of 5 cm and underground length unknown due to its habit (all aproximate values).

Generation length (yr): 0

Dependency of single sp?: No

Ecology and traits (narrative): *Nototriche jamesonii* is a plant that has a prostrate shrub habit, being recorded only in sandy soils with scattered vegetation, characteristic of the superpáramo ecosystem. This species has the highest altitude collection record, reported almost at 5000 m close to Whymper's Refuge in Chimborazo volcano. It grows on an ecosystem that possesses extreme climatic conditions such as strong winds, low temperatures, lack of precipitation and intense solar radiation.

#### Threats

Justification for threats: *Nototriche jamesonii* is exposed to several anthropogenic threats, most of them relating to tourism and recreation. These activities involve trampling of vegetation along trecking trails and the natural landscape, the construction of roads and infrastructure that interfere with superpáramo ecosystem, leading to pollution. In addition, as the species grows around Ecuador’s largest volcano, it is highly vulnerable to climate change, experiencing constant abnormal warming year by year, as well as intense climatic fluctuations as a result (Morueta-Holme et al. 2015).

##### Threats

Threat type: Ongoing

Threats: 1.3. Residential & commercial development - Tourism & recreation areas3.3. Energy production & mining - Renewable energy4.1. Transportation & service corridors - Roads & railroads6.1. Human intrusions & disturbance - Recreational activities9.4. Pollution - Garbage & solid waste10.1. Geological events - Volcanoes11.3. Climate change & severe weather - Temperature extremes11.5. Climate change & severe weather - Other impacts

##### Threats

Threat type: Future

Threats: 11.1. Climate change & severe weather - Habitat shifting & alteration11.2. Climate change & severe weather - Droughts11.3. Climate change & severe weather - Temperature extremes11.5. Climate change & severe weather - Other impacts

#### Conservation

Justification for conservation actions: There is not a specific action to conserve *N. jamesonii*, but the greatest population recorded at Chimborazo volcano grows inside the Reserva Faunísitica Chimborazo.

##### Conservation actions

Conservation action type: In Place

Conservation actions: 1.1. Land/water protection - Site/area protection

##### Conservation actions

Conservation action type: Needed

Conservation actions: 2.3. Land/water management - Habitat & natural process restoration4.3. Education & awareness - Awareness & communications

### Nototriche phyllanthos

#### Species information

Scientific name: Nototriche phyllanthos

Species authority: (Cav.) A.W. Hill

Synonyms:

≡ *Sida phyllanthos* ≡ *Malvastrum phyllanthos* (Cav.) A. Gray ≡ *Malveopsis phyllanthos*

= *Sida pichinchensis* ≡ *Malvastrum pichinchense* ≡ *Malveopsis pichinchensis*

= Sida pichinchensis var. angusta A.W.Hill

= *Sida saxifraga* Bonpl.

Common names: No common names have been registered for this species.

Kingdom: Plantae

Phylum: Tracheophyta

Class: Magnoliopsida

Order: Malvales

Family: Malvaceae

Taxonomic notes: *Nototriche phyllanthos* is the first species of the genus described for Ecuador. It can be easily recognised by the combination of the following traits: palmati-lobed leaves covered with trichomes only on the adaxial surface of the leaf blade, abaxial surface glabrous, long triangular stipules, free petals and a prostrate shrub habit.

Figure(s) or Photo(s): Fig. 9

Region for assessment: Global

#### Editor & Reviewers

##### Reviewers

Reviewers: N/A

##### Editor

Editor: Trujillo EJ, Muriel P

#### Geographic range

Biogeographic realm: Neotropical

Countries: Ecuador

Map of records (image): Fig. 10

Map of records (Google Earth): None

Basis of EOO and AOO: Observed

Basis (narrative): The extent of occurrence (EOO) is ca. 11,075.53 km and the maximum actual area of occupancy (AOO) is 108.00 km^2^.

Min Elevation/Depth (m): 4200

Max Elevation/Depth (m): 4660

Range description: *Nototriche phyllanthos* is an endemic species from Ecuador that can grow from the central to the northern part of the country. The species is mostly recorded in some northern páramos, such as the ones around Antisana and Pichincha volcanoes, having its highest abundance of individuals in the latter one.

#### Extent of occurrence

EOO (km2): 11,075.53

Trend: Unknown

Justification for trend: There is not enough evidence to establish a concrete trend about the extent of occurrence for the species. However, it is necessary to remark that *N. phyllanthos* grows in threatened and fragmented habitats like the páramos of Ecuador.

Causes ceased?: Unknown

Causes understood?: Unknown

Causes reversible?: Unknown

Extreme fluctuations?: Unknown

#### Area of occupancy

Trend: Stable

Justification for trend: This species has a high abundance in all the localities where it is found, as the individuals like to grow next to each other.

Causes ceased?: No

Causes understood?: Yes

Causes reversible?: Unknown

Extreme fluctuations?: Unknown

AOO (km2): 108.00

#### Locations

Number of locations: 6

Justification for number of locations: This species has been reported at different páramos, most of them belonging to the northern part of Ecuador. *Nototriche phyllanthos* can be found around the Pichincha, Antisana and Cotopaxi volcanoes, Quispicacha summit in Tungurahua, in some parts around Chimborazo and also at Lagunas de Ozogoche.

Trend: Unknown

Justification for trend: This species grows in a variety of localities. Nowadays, we know six locations; however, sampling in other páramos is needed to establish a trend, for example, Quilindaña summit, Ilinizas volcanoes and Altar volcano. These localities have ecosystems that probably can be optimal for *N. phyllanthos*.

Extreme fluctuations?: Unknown

#### Population

Number of individuals: Unknown

Trend: Unknown

Justification for trend: The number of individuals of *N. phyllanthos* cannot be estimated since they grow so closely, but the species has a high abundance in northern páramos. More evidence is needed to establish a trend in the populations.

Causes ceased?: Unknown

Causes understood?: Unknown

Causes reversible?: Unknown

Extreme fluctuations?: Unknown

#### Subpopulations

Number of subpopulations: Unknown

Trend: Unknown

Justification for trend: As we do not know with clarity the entire population of *N. phyllanthos*, the subpopulations cannot be analysed. We need more sampling and evidence to establish a trend.

Extreme fluctuations?: Unknown

Severe fragmentation?: Yes

#### Habitat

System: Terrestrial

Habitat specialist: Yes

Habitat (narrative): The habitat where *N. phyllanthos* grows is characterised by humid conditions with abundance of herbaceous vegetation, cushions and subshrubs. However, this species can also be found in dry zones with scattered vegetation, near to the volcanoes it inhabits.

Trend in extent, area or quality?: Decline (observed)

Justification for trend: As many other páramos in Ecuador, the habitat is in a constant decline in quality and area due to the threats reported above and the historical land usage that people enforce on them.

##### Habitat

Habitat importance: Major Importance

Habitats: 6. Rocky areas (e.g. inland cliffs, mountain peaks)

#### Ecology

Size: Not well defined, it is a species with high phenotypic plasticity.

Generation length (yr): 0

Dependency of single sp?: No

Ecology and traits (narrative): *Nototriche phyllanthos* can be found growing as a prostrate shrub, with extremely variable traits like the size of vegetative and reproductive structures, length of the roots, rosette compactation etc. This species is comfortable growing beside another individuals from the same species or other plants. Additionally, it can be found on dry zones, but in those cases, the compactionn of rosettes is higher than on the individuals that grow in humid soils.

#### Threats

Justification for threats: This species grows in páramo habitats located very close to urban areas, as shown by the individuals recorded around Pichincha volcano. Several threats have been identified that affect this species, like development of urban areas, housing, argiculture and farming and trampling, that lead to the construction of roads and other anthropogenic structures. The mentioned anthropogenic presence also results in the production of garbage and different kinds of waste that pollute the ecosystem where *N. phyllanthos* live. In addition, energy conducting infrastructure like antennas were placed on its habitat, carrying an impact on the flora, in association with the introduction of heavy machinery. Finally, as a high-elevation species, it is vulnerable to the impacts of climate change, with rising temperatures threatening these characteristically cold páramo ecosystems.

##### Threats

Threat type: Ongoing

Threats: 1.1. Residential & commercial development - Housing & urban areas1.3. Residential & commercial development - Tourism & recreation areas2.1.2. Agriculture & aquaculture - Annual & perennial non-timber crops - Small-holder farming3.3. Energy production & mining - Renewable energy4.1. Transportation & service corridors - Roads & railroads6.1. Human intrusions & disturbance - Recreational activities7.3. Natural system modifications - Other ecosystem modifications9.1.3. Pollution - Domestic & urban waste water - Type Unknown/Unrecorded9.3.4. Pollution - Agricultural & forestry effluents - Type Unknown/Unrecorded9.4. Pollution - Garbage & solid waste11.3. Climate change & severe weather - Temperature extremes11.5. Climate change & severe weather - Other impacts

##### Threats

Threat type: Future

Threats: 11.1. Climate change & severe weather - Habitat shifting & alteration11.2. Climate change & severe weather - Droughts11.3. Climate change & severe weather - Temperature extremes11.5. Climate change & severe weather - Other impacts

#### Conservation

Justification for conservation actions: There is not an specific action to conserve *N. phyllanthos*, but some of the populations that were visited are under protected areas or reserves. However, the greatest population of the species found at Pichincha volcano is not under any protecting scheme, being vulnerable in the face of the threats mentioned above.

##### Conservation actions

Conservation action type: In Place

Conservation actions: 1.1. Land/water protection - Site/area protection

##### Conservation actions

Conservation action type: Needed

Conservation actions: 2.3. Land/water management - Habitat & natural process restoration4.3. Education & awareness - Awareness & communications

## UICN Red List Assessments

***Nototriche antisanensis***

We obtained an EOO of 0.03 km^2^ and an AOO of 8.00 km^2^ for *N. antisanensis*, both below the thresholds for the Critically Endangered category. Considering the level of fragmentation, the number of threats and the population size (< 50 mature individuals), we assess the global threat category of *N. antisanensis* as **Critically Endangered (CR)** under criteria **B2ab(iii) + D** of the IUCN (2012) maintaining the category provisionally established by Trujillo et al. (2025b).

***Nototriche ecuadoriensis***

Based on its EOO (243.50 km^2^) and its AOO (16.00 km^2^), *N. ecuadoriensis* is below the threshold of the category Endangered (EN), which is the category reported in Montúfar and Pitman (2004a). However, the observed population reduction, the low number of individuals (unknown, but predicted less than 50 mature individuals for each subpopulation), the high fragmentation of the subpopulations and the existing threats and impact of climate change are variables that must be taken into account in the evaluation. Considering all the mentioned data for the taxon, *N. ecuadoriensis* can be classified as **Critically Endangered (CR)** under criterion **C2a(i)** of the IUCN (2012).

***Nototriche hartwegii***

Considering the number and type of threats, most of them caused by anthropogenic intervention, number of populations, subpopulations, the habitat where it lives and the extremely fragmented distribution the species has, adding the values of EOO (10492.00 km^2^) and AOO (28.00 km^2^), we assess the global threatened category of *N. hartwegii* as **Endangered (EN)** under criteron **B2ab(iii)** of the IUCN (2012), mantaining the one established by León-Yánez et al. (2011) and Trujillo et al. (2025a).

***Nototriche jamesonii***

In a previous assessment (Montúfar and Pitman 2004b), *N. jamesonii* was categorised as Low Concern (LC); however, when these analyes were made, the herbarium specimens were not properly identified and an exhaustive field sampling of the genus in the country was lacking. Nowadays, we know that the populations of *Notoriche jamesonii* are restricted to the central Andes of the country and have their highest concentration around Chimborazo volcano. With an EOO and AOO of 712.15 km^2^ and 48.00 km^2^, respectively and considering the mentioned threats, we suggest to consider this species under the category of **Endangered (EN)** under **B1ab(iii)** of the IUCN (2012).

***Nototriche phyllanthos***

Considering the EOO (13,363.12 km) and the AOO (108.00 km^2^), in addition to the actual knowledge about the distribution of the species and the threats it suffers, we consider pertinent change to the initial status of Least Concern (LC) established in previous conservation assessments and suggest to place *Nototriche phyllanthos* below the category **Vulnerable (VU)** under criterion **B2b(iii)** of the IUCN (2012).

UICN Red List Assessments

## Figures and Tables

**Figure 1.**
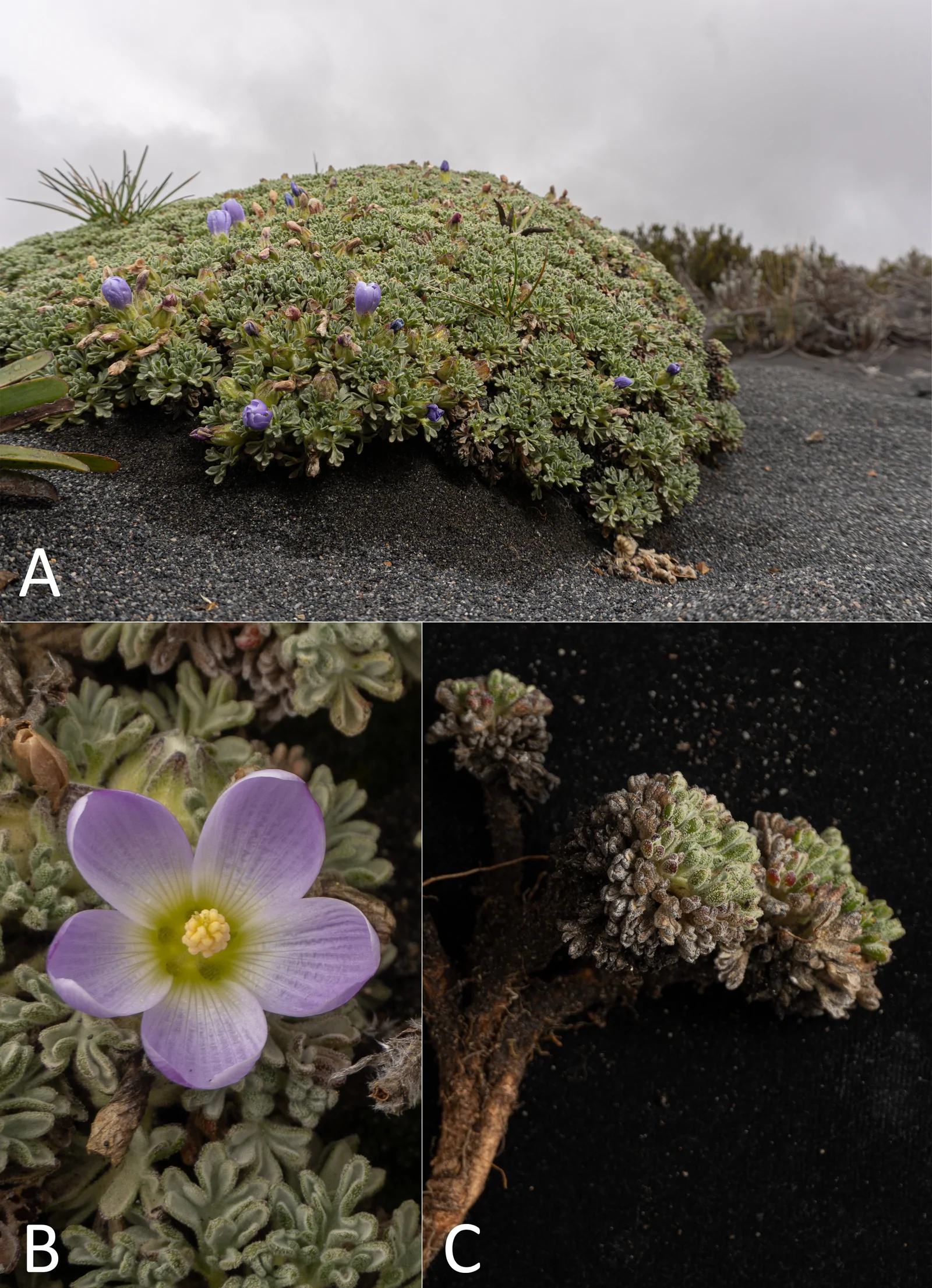
*Nototriche antisanensis* E.J. Trujillo, Muriel, Espinel-Ortiz & Romol. **A** Species habit; **B** Close-up top view of the flower; **C** Leaves in rosette. Photographs: Erick Troncoso-López.

**Figure 2.**
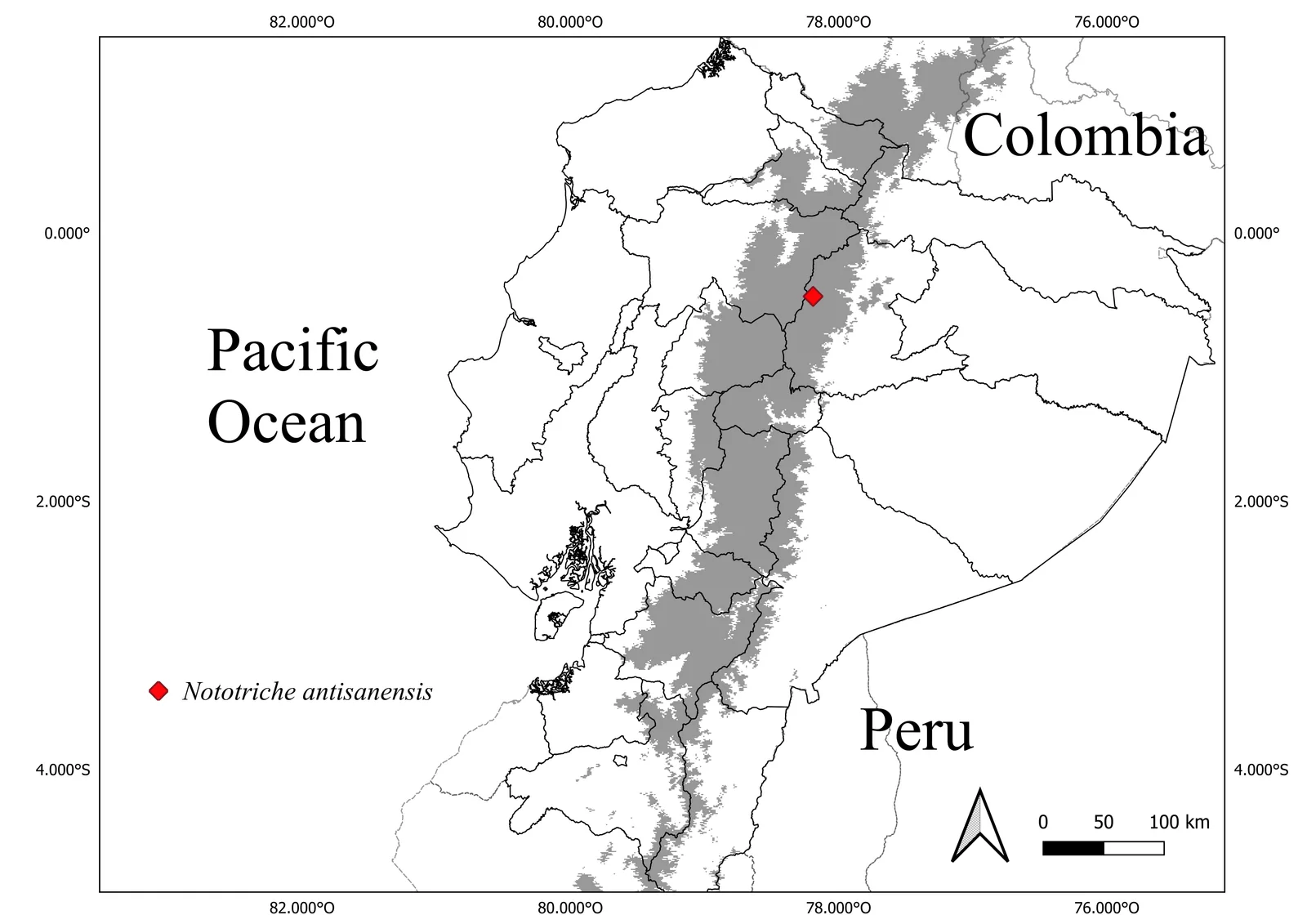
Distribution of *Nototriche antisanensis* in the Ecuadorian Andes (red diamond), with only one population in the north-western slopes of Antisana volcano.

**Figure 3.**
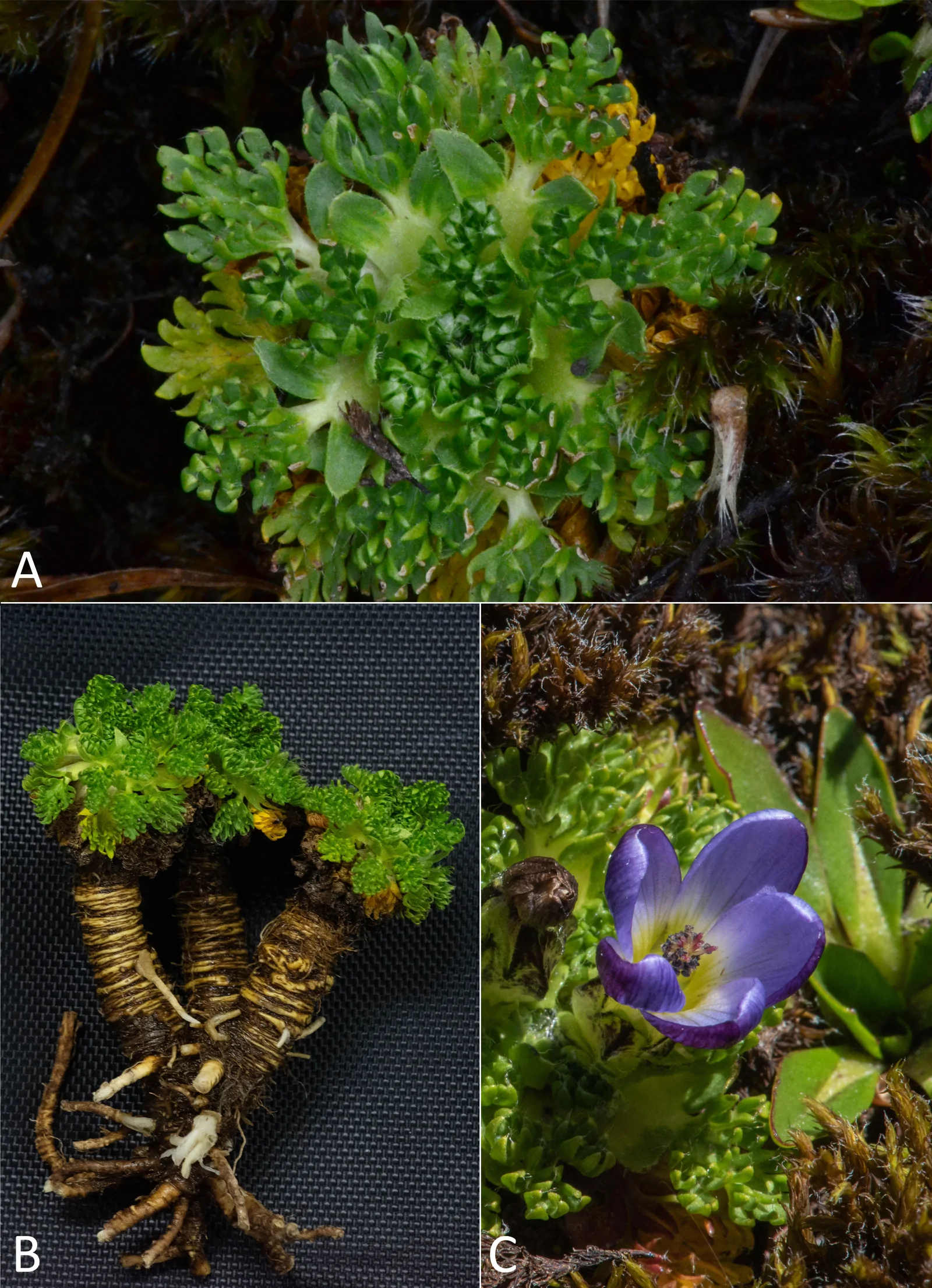
*Nototriche ecuadoriensis* Fryxell. **A** Leaves showing lobes and stipules; **B** Habit; **C** Flower. Photographs: **A & B** Emilio J. Trujillo; **C** Ricardo Jaramillo.

**Figure 4.**
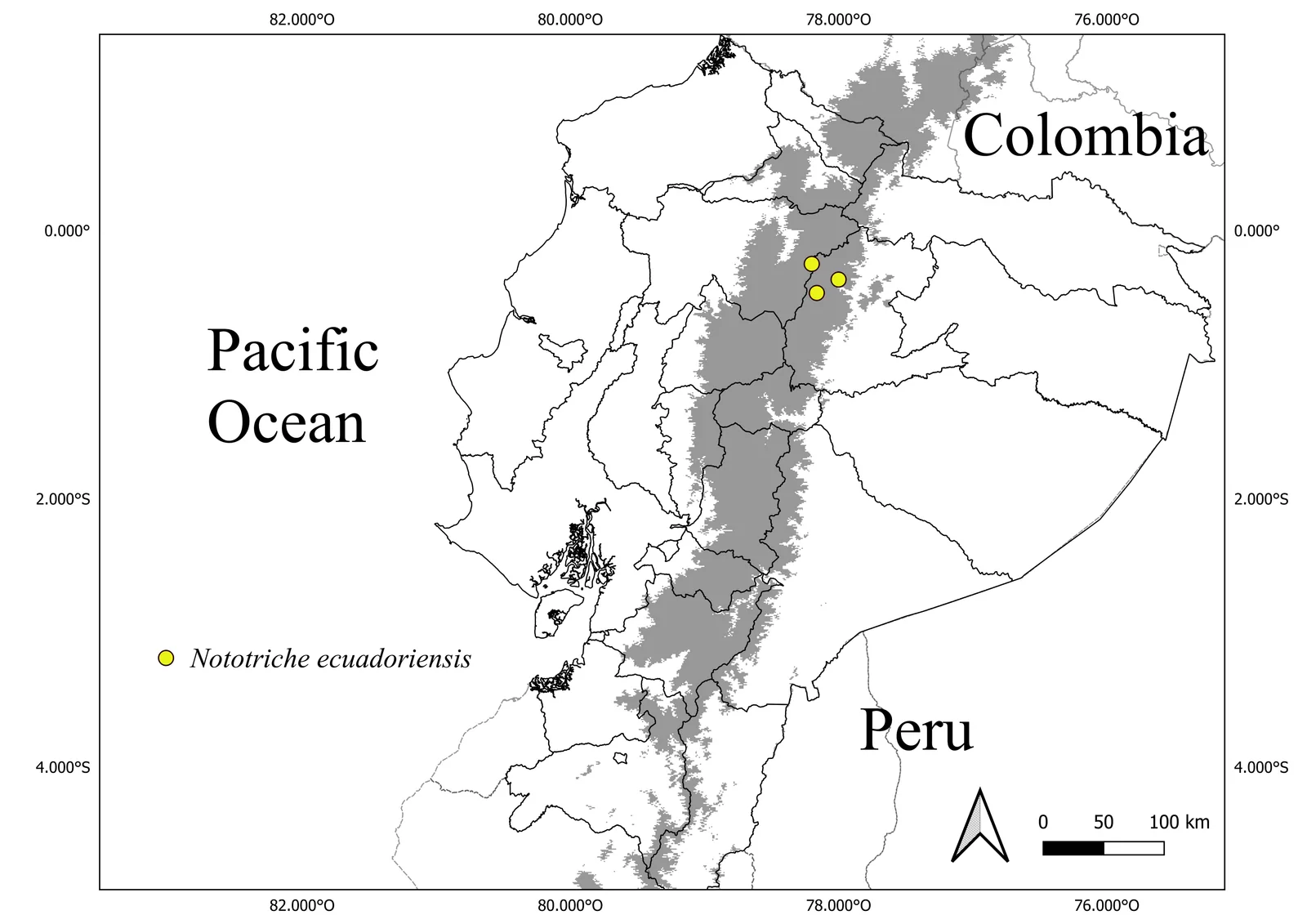
Distribution of *Nototriche ecuadoriensis* in the Andes of Ecuador (yellow dots), with three subpopulations, located all at the north-eastern Andean mountain range.

**Figure 5.**
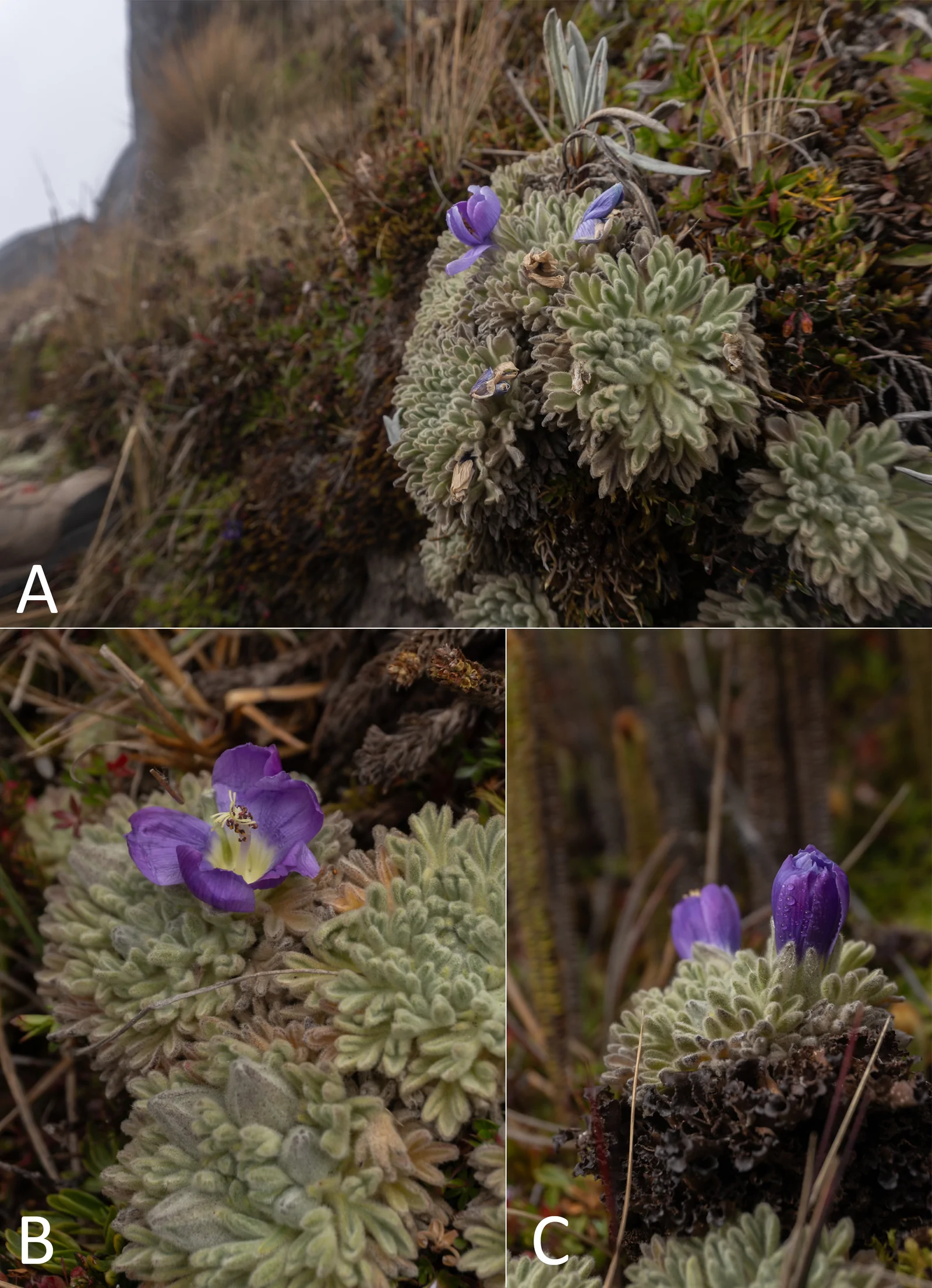
*Nototriche hartwegii* A.W.Hill. **A** Species habit, showing a cushion-forming individual; **B** Flower; **C** Individual rosette. Photographs: Erick Troncoso-López.

**Figure 6.**
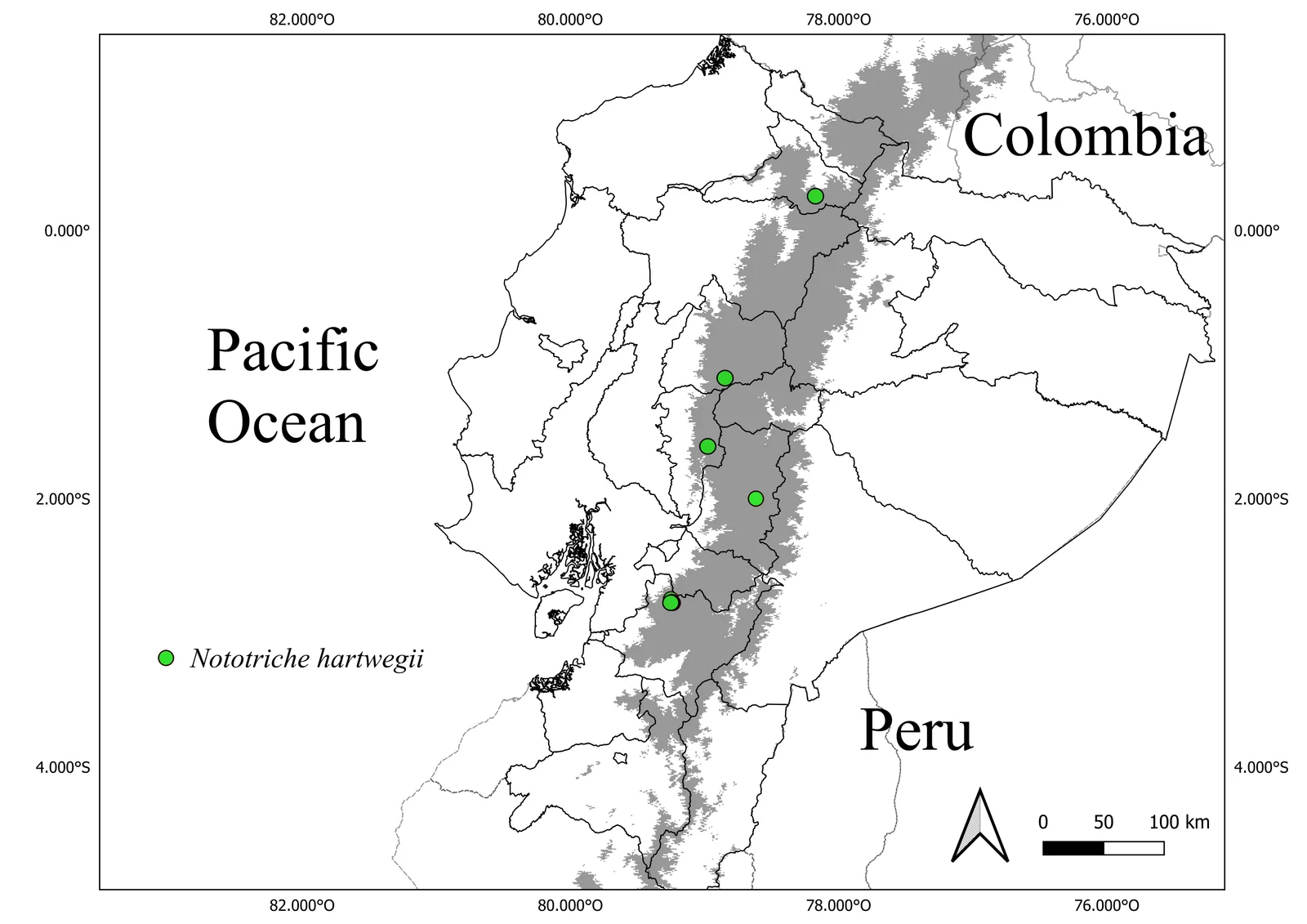
Distribution of *Nototriche hartwegii* in the Ecuadorian Andes (green dots); there are five known localities distributed between the southern, central and northern parts of the country.

**Figure 7.**
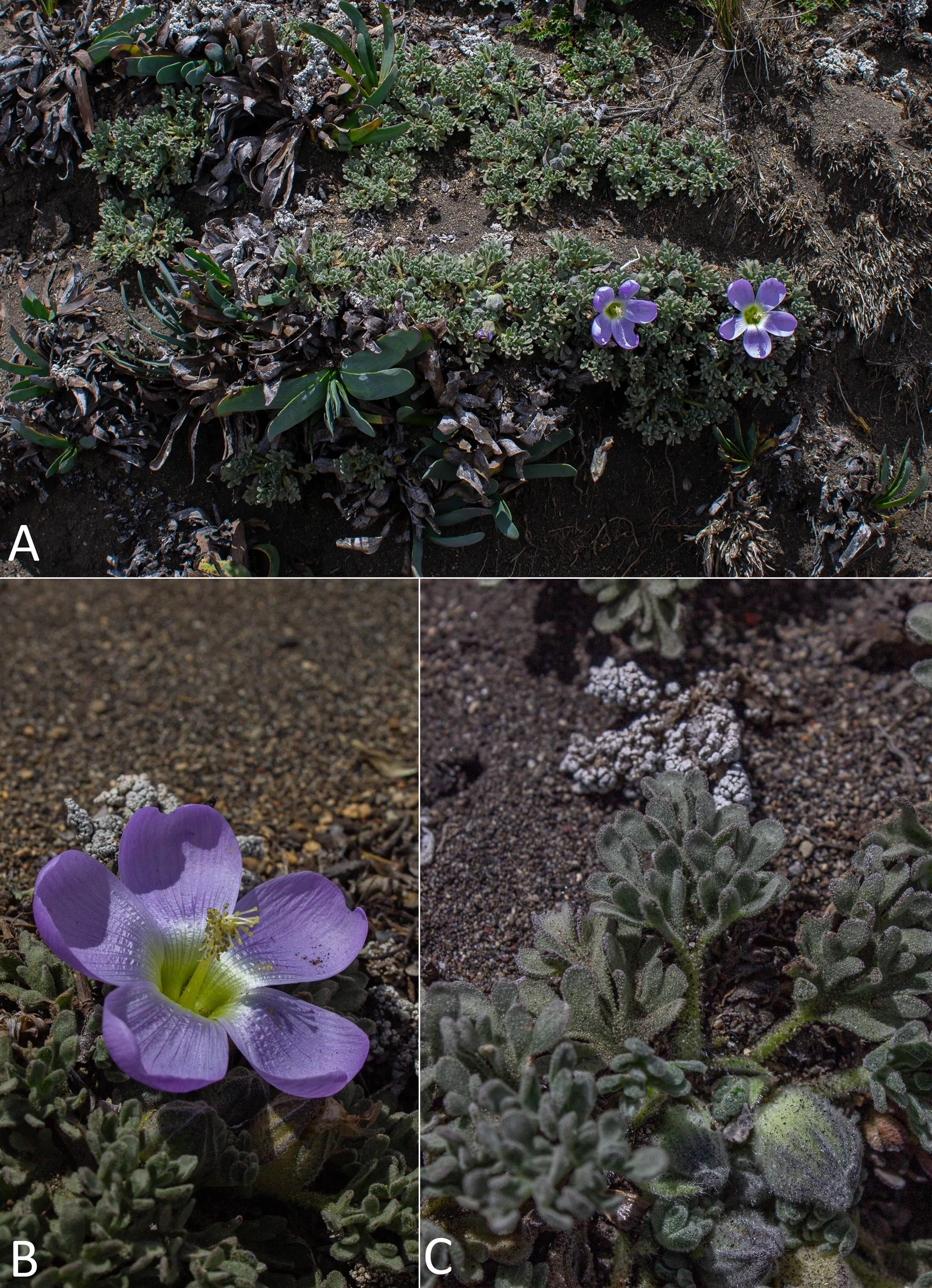
*Nototriche jamesonii* A.W.Hill. **A** Habit; **B** Flower; **C** Close-up of the leaves. Photographs: Emilio J. Trujillo.

**Figure 8.**
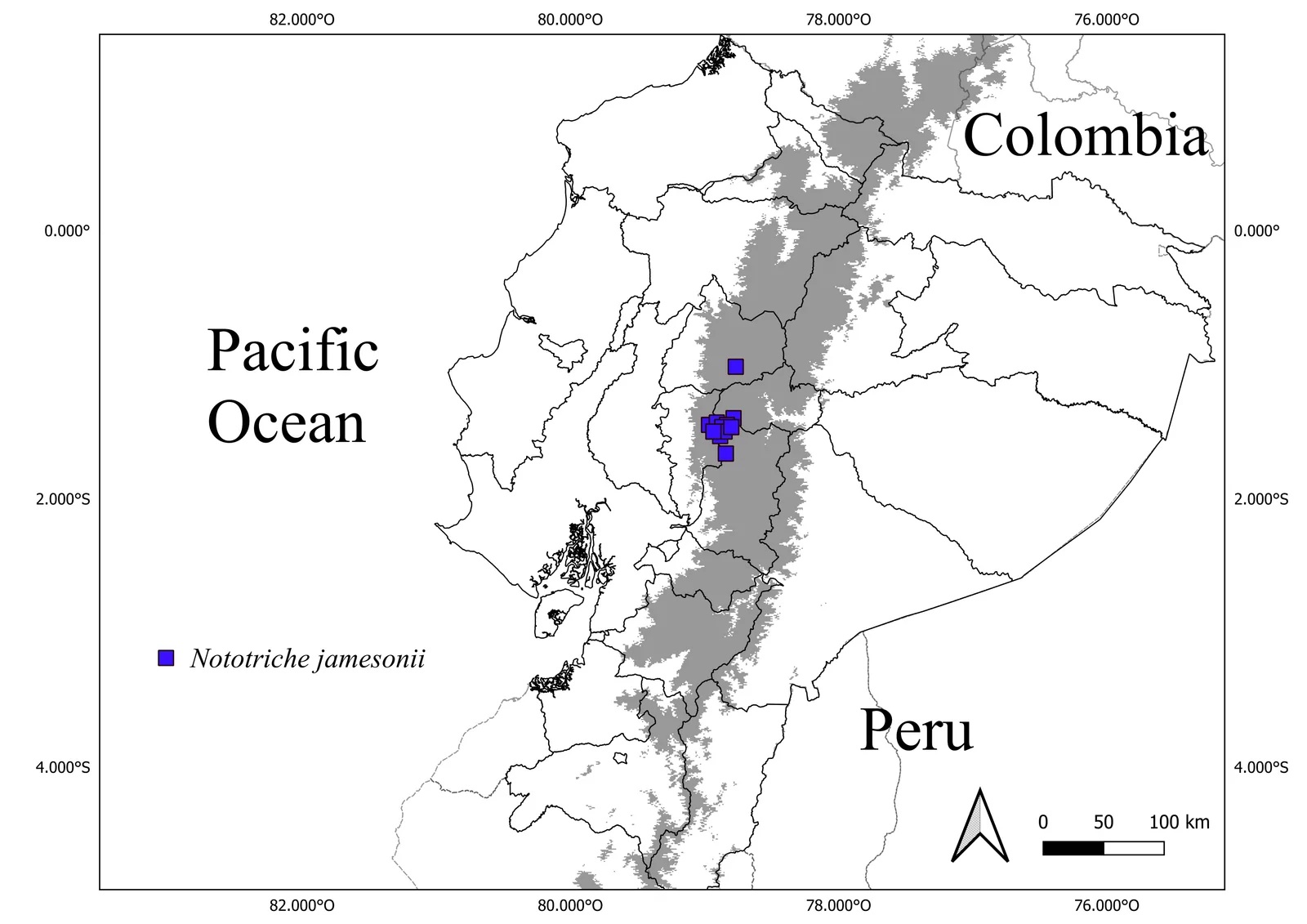
Distribution of *Nototriche jamesonii* in the Ecuadorian Andes (blue squares), with two known localities.

**Figure 9.**
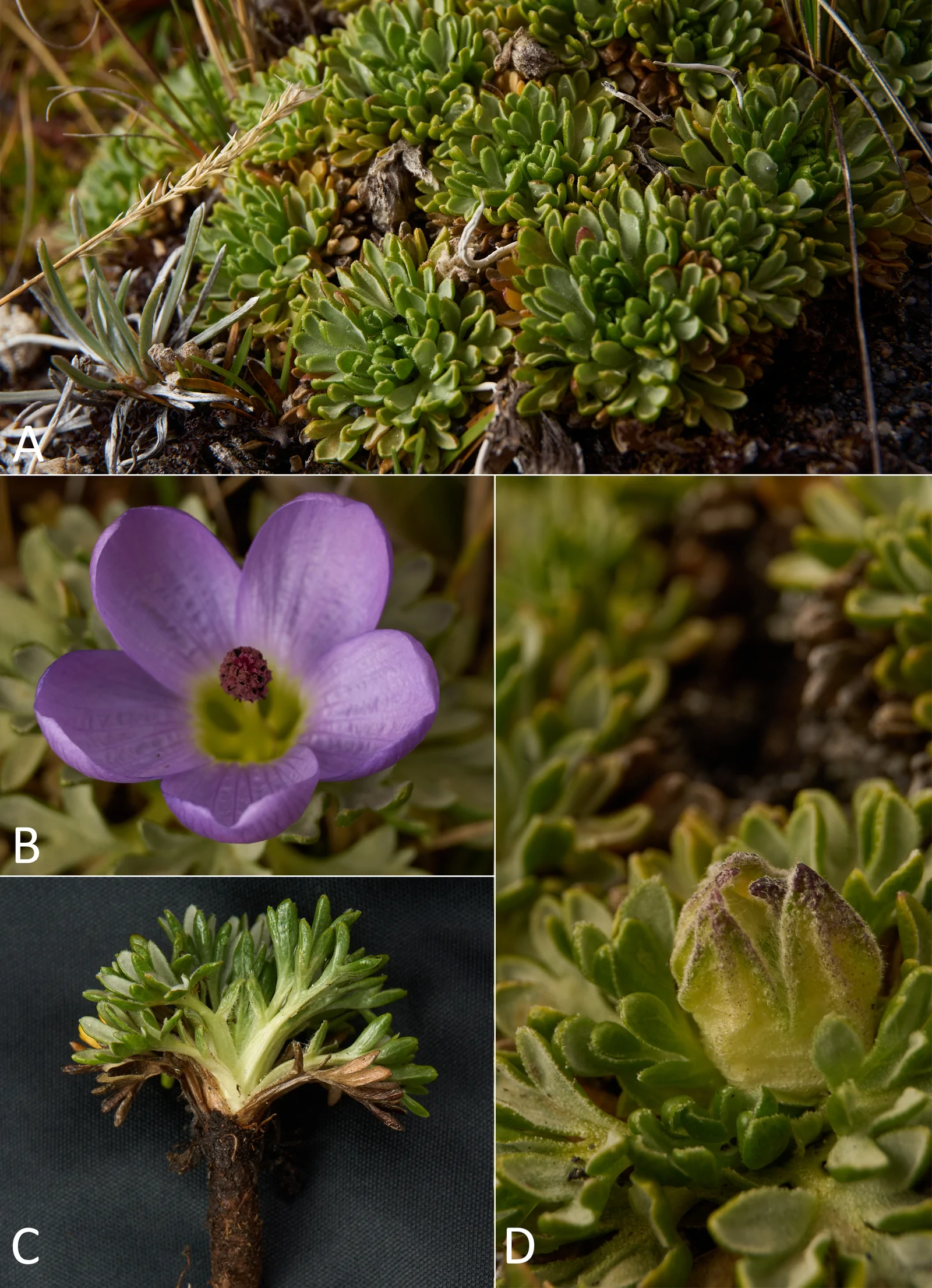
*Nototriche phyllanthos* (Cav.) A.W.Hill. **A** Habit; **B** Flower; **C** Close-up of the leaves showing the rosette; **D** Flower button. Photographs: Erick Troncoso-López.

**Figure 10.**
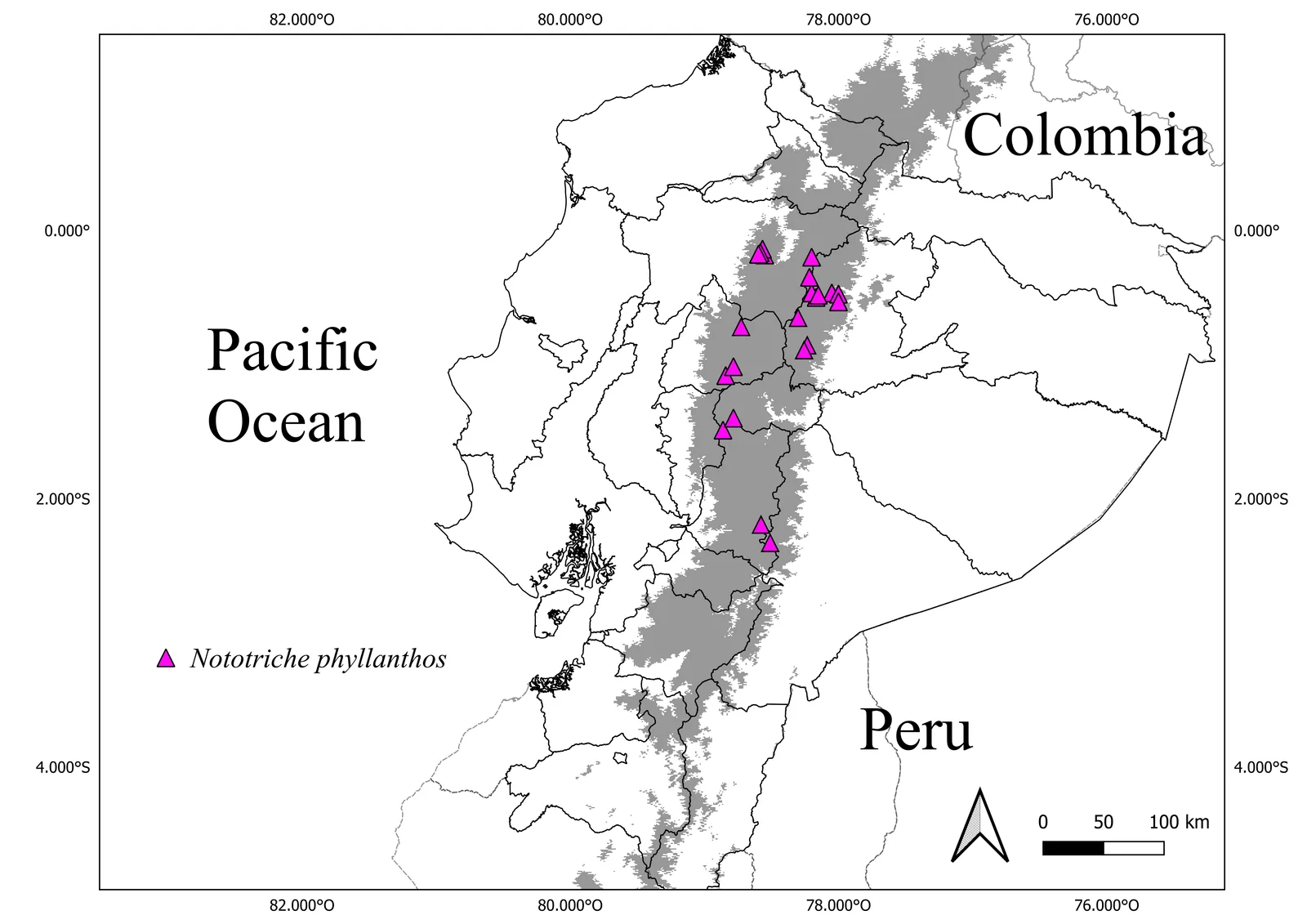
Distribution of *Nototriche phyllanthos* in the Andes of Ecuador (pink triangles), well distributed in the northern and central areas of the country.

**Table 1.** Summary of the proposed categories of each *Nototriche* species.

**Species**	**Previously proposed category**	**Publication**	**New proposed category**
*Nototriche antisanensis*	CR (Preliminary)	Trujillo et al. (2025)	CR
*Nototriche ecuadoriensis*	EN	León-Yánez et al. (2011)	CR
*Nototriche hartwegii*	EN	León-Yánez et al. (2011)	EN
*Nototriche jamesonii*	LC	León-Yánez et al. (2011)	EN
*Nototriche phyllanthos*	LC	León-Yánez et al. (2011)	VU

## References

[B13435281] Bachman Steven, Moat Justin, Hill Andrew, de la Torre Javier, Scott Ben (2011). Supporting Red List threat assessments with GeoCAT: geospatial conservation assessment tool. ZooKeys.

[B13435272] Chanco M, Ulloa-Ulloa C (2004). Las especies de *Nototriche* (Malvaceae) de Ecuador. Sida, Contributions to Botany.

[B13435291] Fryxell PA, Harling G, Andresson L (1992). Flora of Ecuador.

[B13584602] Hall Minard, Mothes Patricia, Aguilar Jorge, Bustillos Jorge, Ramón Patricio, Eissen Jean-Philippe, Monzier Michel, Robin Claude, Egred José, Militzer Annemarie, Yepes Hugo (2012). Los peligros volcánicos asociados con el Antisana.

[B13441351] Hill AW (1909). A revision of the genus *Nototriche*, Turcz.. Transactions of the Linnean Society of London. 2nd Series: Botany.

[B13435304] Hofstede R, Mena-Vásconez P, Suárez E (2023). Los páramos del Ecuador: Pasado, presente y futuro.

[B13472403] Hofstede R, Llambí L, Peralvo M, Beltrán K, Calispa M, Mosquera G, Hofstede R, Mena-Vásconez P, Suárez E (2023). Los páramos del Ecuador: Pasado, presente y futuro.

[B13435450] IUCN, UICN (2012). IUCN Species Survival Commission (Ed.) IUCN Red List Categories and Criteria..

[B13441360] Krapovickas A (1951). Notas cito-taxonómicas en Nototriche (Malvaceae) I. Boletín de la Sociedad Argentina de Botánica.

[B13434690] León-Yánez SC, Valencia R, Pitman N, Endara L, Ulloa-Ulloa C (2011). Libro rojo de las plantas endémicas del Ecuador.

[B13440994] Lomolino M, Riddle B, Brown J (2005). Biogeography.

[B13441071] Mazzei P (2025). El género *Nototriche* Turcz. (Malvoideae: Malvaceae) en el Perú: Revisión Taxonómica y Distribución.

[B14387369] Montúfar R, Pitman N (2004). Nototriche ecuadoriensis.

[B14387378] Montúfar R, Pitman N (2004). Nototriche jamesonii.

[B14188542] Moret P, Muriel P, Jaramillo R, Dangles O (2019). Tableau Physique revisited. Proceedings of the National Academy of Sciences.

[B14256724] Morueta-Holme N, Engemann K, Sandoval-Acuña P, Jonas J. D., Segnitz R. M., Svenning J (2015). Strong upslope shifts in Chimborazo's vegetation over two centuries since Humboldt. Proceedings of the National Academy of Sciences.

[B13441332] Pérez-Escobar OA, Zizka A, Bermúdez M, Meseguer A, Condamine F, Hoorn C, Hooghiemstra H, Pu Y, Bogarín D, Boschman L, Pennington R, Antonelli A, Chomiki G (2022). The Andes through time: evolution and distribution of Andean floras. Trends in Plant Science.

[B14387809] Team QGIS Development (2026). QGIS Geographic Information System.

[B13441047] Romoleroux K, Muriel P, Sklenář P, Ulloa-Ulloa C, Espinel-Ortiz D, Romoleroux C, Hofstede R, Mena-Vásconez P, Suáres E (2023). Los páramos del Ecuador: Pasado, presente y futuro.

[B13472390] Sarmiento G, Vuilleumier F, Monasterio M (1986). High altitude tropical biogeography.

[B13472373] Sklenář P, Kucerová A, Macková J, Macek P (2015). Temporal variation of climate in the high-elevation páramo of Antisana, Ecuador. Geografia Fisica e Dinamica Quaternaria.

[B14387783] Sklenář P, Ramsay PM (2001). Diversity of zonal páramo plant communities in Ecuador.. Diversity and Distributions.

[B14256744] Sklenář P, Romoleroux K, Muriel P, Jaramillo R, Bernardi A, Diazgranados M, Moret P (2021). Distribution changes in páramo plants from the equatorial high Andes in response to increasing temperature and humidity variation since 1880. Alpine Botany.

[B14256735] Trujillo E. J., Romoleroux K, Muriel P (2025). New records of *Nototriche
hartwegii* A.W.Hill (Malvaceae, Malvoideae) in Ecuador: updated distribution and range extension. Checklist.

[B13430438] Trujillo Emilio José, Espinel-Ortiz DA, Romoleroux Katya, Muriel Priscilla (2025). A new species of *Nototriche* (Malvaceae, Malvoideae) from the high Andes of Ecuador. PhytoKeys 261: 1-12..

